# Health Policies Based on Patient Satisfaction: A Bibliometric Study

**DOI:** 10.3390/healthcare9111520

**Published:** 2021-11-08

**Authors:** Mayra Soledad Grasso, María del Carmen Valls Martínez, Alicia Ramírez-Orellana

**Affiliations:** 1 Mediterranean European Center of Economics and Sustainable Development (CIMEDES), University of Almería, 04120 Almería, Spain; mayragrasso21@gmail.com; 2 Department of Economics and Business, University of Almería, 04120 Almería, Spain; aramirez@ual.es

**Keywords:** patient satisfaction, health policy, health system, Scopus, bibliometric analysis, research trends

## Abstract

Healthcare decision-makers increasingly face a changing and ever-evolving landscape, forcing them to formulate public policies based on the results from different scientific investigations. This article evaluates the field of research on patient satisfaction as a basis for health policies. The analysis was carried out with a sample of 621 articles published between 2000 and 2020 in the Scopus database. The world’s largest producer and research co-operator on patient satisfaction and health policy was the United States. However, the most prolific authors, institutions, and journals are of British origin. Regarding the themes, we find that, in economic and management matters, scientific production is scarce. To study the evolution of keywords, we divided the study period into two periods of an equal number of years. In both sub-periods, the keyword “Human” stands out. In the second sub-period, the word “Perception” stands out, which indicates the current attention paid to the patient’s opinion.

## 1. Introduction

The vision of health system quality has evolved over time and no longer includes merely scientific-technical concepts [1] but also perceived quality, that is, the difference between what the clients (patients) expect and what they perceive [2,3]. It is not enough to meet the needs of patients to achieve their satisfaction; it will also be necessary to meet their expectations [4,5]. In the middle of the last century, Koos (1954) [6] and Donabedian (1966) [7] were pioneers in measuring healthcare results based on patient satisfaction. At present, there is still no standardised method to measure satisfaction. On the contrary, the existing bibliography considers different perspectives and methodologies [8,9,10,11]. In addition, it is believed that patient satisfaction encompasses various dimensions such as technique, functionality, infrastructure, interaction, atmosphere, and services [12,13].

Measuring satisfaction means comparing patients’ needs and expectations of medical care with their own experience [14]. The purpose of evaluating patient satisfaction is to identify points for improvement by identifying those needs or expectations of patients that have not yet been satisfied [15,16]. Therefore, identifying the strengths and weaknesses of the performance of health services based on the patient’s expectations will be an effective tool for the management and formulation of public policies [17].

The three basic expectation models that have been formulated are the contrast model, the assimilation model, and the assimilation-contrast model. The first assesses the discrepancy between patient expectations and the health system’s performance [18]. The second understands that when there are differences between expectations and reality, the consumer (patient in the healthcare system) adjusts their expectations to reality. Namely, the patient tends to decrease dissonance to maintain coherence between multiple cognitions [19]. Finally, there is an “acceptance circle” in the assimilation-contrast model when the differences are within the acceptable limits. It is assimilated when it is within limits, but when it exceeds them, the contrast theory applies [20].

Patient-centred care primarily implies that the patient is respected and understood [21]. Harvey Picker pioneered the study of patient-centred care, and his institute became the first to collect information on user perceptions of the healthcare system [22]. In Western Europe and North America, it is already a fact that patients play an active role in health services planning and development [23]. This involvement leads to better health outcomes because of its effect on patient satisfaction [4,24] and the health system’s quality [25,26,27].

The European Commission created the European Core Health Indicators (ECHIs), which are health indicators of the European Union whose objective is to obtain comparable and reliable data to contribute to the production of policies. The data emanating from these indicators will give an indication of the appropriate health policies to apply [28].

On the other hand, politics denotes power and conflict between the parties involved, called stakeholders [29,30]. It also includes the strategies used to solve this problem [31]. Health policies will have a direct impact on the experience of patients.

The stakeholders of the health system can be synthesised into three groups. First, healthcare providers are made up of health professionals such as doctors, nurses, etc. This group will claim the best health outcomes regardless of cost. Second, State health policymakers and their professional health advisors and researchers strive for an efficient health system. Some authors even believe that this group ignored or repressed research in response [32] to pressures generated by budgets, electoral campaigns, and social crises, among other things [33]. Lastly, the patients have repressed their interests because they are underrepresented in policy formulation [34].

A study conducted in Sweden revealed that most patients do not accept resource constraints regarding healthcare [35]. This fact will generate greater disagreements.

The World Health Report 2000 [1] indicates that the main goals of the health system are good health, equity of financial contribution, and capacity to respond to the expectations of the population. For all these reasons, this work aims to analyse all scientific production on patient satisfaction with the health system, which is the basis for determining public policies. Later, in 2015, the 2030 Agenda was adopted by the United Nations, where it committed the signatory countries to promote global health [36]. Although this objective is expressly detailed in “Goal 3”, the reality is that it is a cross-cutting issue throughout the 2030 Agenda [37].

The importance of applying good health policies lies in the fact that they influence the quality of life of the current population and condition future possibilities. From a health point of view, it can affect the population’s life expectancy [38,39], and from an economic point of view, it can affect the number of people working in the labour force [40]. In other words, the population’s health status will significantly impact the robustness of the country’s economy. Quality healthcare available to the majority of the country’s population is essential for the country’s growth as this will allow a balance between birth and death rates and a low incidence of diseases [41].

Seen from another point of view, the extension of the life expectancy of the people, and the improvement of their quality of life, could be an inconvenience for the health system of a country that offers its population universal access to medical care [29,42,43]. We highlight the importance of agents understanding political processes and implementing good health policies because they will be the ones who can contribute to the continuous improvement of the services provided [44,45]. The latter takes the premise that patient satisfaction is a strong indicator of the quality of health services [14,24,46,47,48,49].

In recent years, countries’ spending on health matters increased more than the increase in the gross domestic product (GDP) in most countries that belong to the Organization for Economic Cooperation and Development (OECD) [29,43]. Developed countries, on average, allocate between 8% and 10% of their GDP to finance health [50]. However, for example, Spain reduced its healthcare budget after the 2008 crisis by approximately 14% [51]. The key will then be to improve the population health with the minimum use of possible resources, that is, being efficient, understanding that quality and efficiency are not disjunctive concepts.

This paper offers a review of the literature regarding the public politics formulation based on patient satisfaction. A bibliometric analysis was carried out starting from a sample of 971 documents that, after selecting only the articles from 2000 onwards (excluding the year 2021 for not being complete), was reduced to 621 articles. With this research, we were able to study the evolution of knowledge on the subject in question and identify the most prolific authors and the most solid collaborations between countries, among other things.

The OECD defined bibliometrics as a tool to analyse the state of literature and technology with a certain degree of specialisation [52]. Bibliometric studies that refer to the health system can be observed in the bibliography, but not those that specifically treat patient satisfaction as the basis for public policy formulations.

A previous investigation revealed that Europe contributes approximately one-third of the world’s scientific production related to public health [53]. Additionally, we can find bibliometric studies that deal with articles that evaluate the quality of health services [54] or the existing institutional commitment in health organisations [55]. There are also bibliometric articles on health economics [56] and even the particular effect of telemedicine on patient satisfaction [57], along with the study of scientific activity on a specific disease [58]. Our study goes further because its objective is to study the scientific production on patient satisfaction as a basis for formulating public policies.

The objective of the health system will be to improve the health of the population, to which policymakers will need to analyse patients’ perceptions of it [59]. Scientific results constitute an input capable of transforming reality and/or solving problems [60]. Furthermore, it is indisputable that the product of health research must incorporate political content. The latter is because, although science can find significant findings on improving the population’s health, it will be the political actors who will be able to translate them into reality [45]. Accordingly, scientists are increasingly required to demonstrate the politically relevant benefits of their findings [33,61].

The rest of the article is organised as follows: The data and methodology are presented in Section 2. Section 3 shows the results, and the discussion is presented in Section 4.

## 2. Materials and Methods

This research uses bibliometrics to study and analyse scientific activity in the health policy formulation field based on patient satisfaction. Bibliometrics is a sub-discipline within the information sciences that studies the collective behaviour of facts in the informational-documentary field [62]. Based on mathematical and statistical techniques, bibliometrics studies different aspects of scientific activity [63,64]. This methodology can be used to analyse different elements such as the international dimension of the research, the relationship between different units of analysis, and co-authorship analysis [65]. In other words, it will analyse activity indicators, structural indicators, and impact indicators [66]. Methodologically, we could assimilate scientific production with a company's production that must evaluate its inputs and results [65].

Currently, there are different online bibliographic databases, but they do not cover the scientific field in the same way [65]. The central databases are Scopus and Web of Science (WoS). Scopus was chosen for this work because it covers a broader set of journals [67]. Indeed, 84% of WoS articles are in Scopus [68].

**Figure 1:**
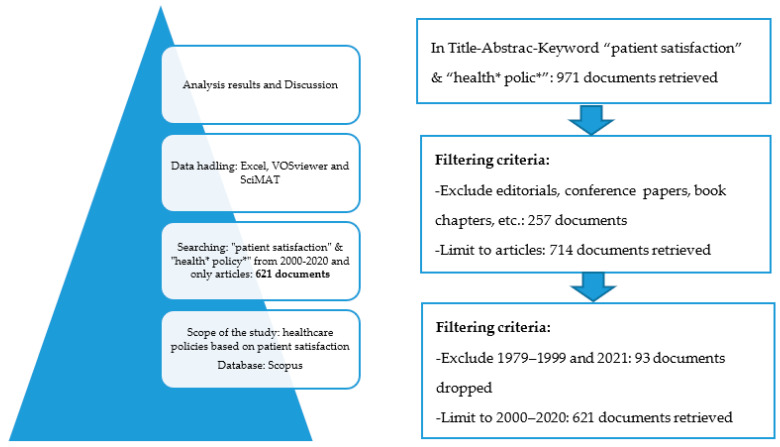
The methodology followed in the selection and processing of information.

The investigation was divided into four steps: (1) the definition of the field of study and the database to be used, (2) research criteria adjustment, (3) codification of recovered material, and (4) analysis of the results and discussion. Figure 1 describes the methodology followed for the selection and processing of the information.

As mentioned above, and as expressed in the article’s title, our objective was to analyse the existing literature on the formulation of health policies based on patient satisfaction. Public managers are faced with investment choices due to limited budgets and increasing costs due to technology and an ageing population caused by longer life expectancy. On the other hand, patient satisfaction is increasingly used to assess the quality of the healthcare system. Consequently, the following parameters were used to retrieve the search: Title-Abstract-Keyword (“patient satisfaction” and “health* policy*”), and 971 documents were obtained. The investigation was carried out in April 2021, and the study period was from 2000 to 2020. Considering that health policies have to face different challenges than in the past, with a larger and older population, advances in costly means of therapy and diagnosis, increasing quality demands from the patient, etc., we decided to focus the study on the current era. The year 2021 was not included so that only entire years would be compared. This time restriction narrowed the search to 714 documents. In addition, we limited the study to scientific articles, excluding reviews, conference papers, book chapters, etc., because they tend to have repeated content, while articles present the research novelties. We excluded the documents that did not meet the agreed criteria, and, finally, we followed the analysis with 621 articles.

The data were managed with Excel, VOSviewer, and SciMAT. On the one hand, VOSViewer software (University of Leiden, Leiden, Holland allows graphical representations of the data and its relationships, favouring the interpretation and understanding of the information [69,70,71]. On the other hand, SciMAT (University of Granada, Granada, Spain) is a tool used to analyse scientific maps in a longitudinal framework that allows measuring the centrality and density of each research topic using strategic diagrams [71]. Different authors have already used this tool in areas such as tourism [72,73], sustainability [74,75], business [76], and education [77,78], among others, with the intention of finding associations and new research trends.

Strategy diagrams measure two dimensions: density and centrality. Centrality is defined as the degree of the interaction of different research topics, and density is the internal strength value of the research topic object of study [74]. Those themes with a high density and centrality will be called motor themes. The opposites will be called emerging or disappeared themes because they are marginal and underdeveloped themes. Although they are well developed internally, those isolated from other issues will be peripheral [71]. Finally, the basic, general, and transversal topics are those topics that are important for the scientific field but that are not well developed [69]. In addition, it is possible include a third dimension in the diagram, which is displayed in the volume of the sphere and could represent different bibliometric indicators (number of citations, number of documents and h-index). Two periods were determined to carry out the analysis: the first period of initial development (2000 to 2010) and a second period for the last ten years (2011 to 2020) to study trends within the study area. For each period analysed, using the SciMAT tool, two strategic maps were constructed, through the measures of centrality and density, using the methodology proposed by Cobo et al. (2012).

## 3. Results

In order to suggest or encourage future lines of research, to determine which areas are sufficiently investigated and which require greater penetration, it is necessary to investigate the scientific production of the researchers. It can also be helpful to study each line of research based on a country, an author, or even a specific institution.

This section of the article is divided into two parts. The first part twill evaluate the evolution of scientific production in terms of published articles, productive countries, and the number of citations per article, among other things. The second part will evaluate the content of scientific production to define topics that should promote further research. Table 1 shows a summary of the data used to carry out the bibliometric study extracted from Scopus. These data are divided into two groups, defined according to a period of 10 years each.

### 3.1. Descriptive Analysis

#### 3.1.1. Evolution of Scientific Production

**Figure 2:**
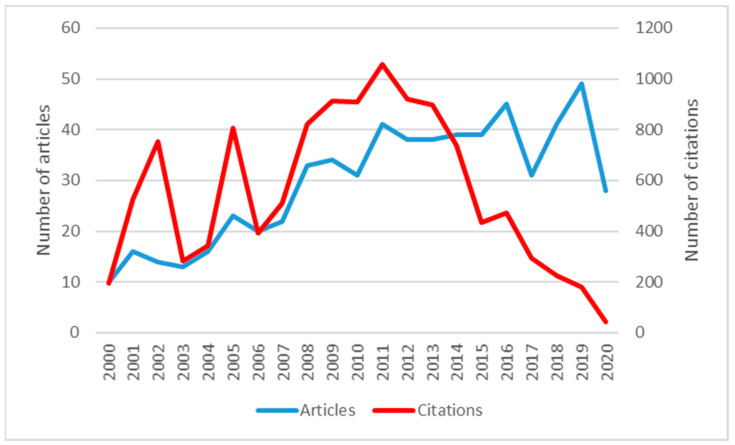
Evolution over time of published articles and total citations.

The 621 articles that make up the sample selected were published between 2000 and 2020. Figure 2 shows that Patient Satisfaction and Health Policy (PS and HP) has had an increasing global trend of scientific production.

Despite the growing trend, two very pronounced decreases can be observed in 2017 and 2020. This latest decrease occurs immediately after the number of publications climbs to its peak in 2019. Therefore, the decline may be caused by the COVID 19 Health Crisis, and it is likely to be an isolated event.

A total of 2672 authors wrote all the articles included in the sample. We can see that 62.64% of the total has been published during the last ten years, so it can be deduced that it is an emerging issue. The citations reached their peak in 2011 with 1059.

With respect to the number of citations, it can be observed that those years with the highest number of citations correspond to those with the highest scientific production. In the articles corresponding to the last six years, the number of citations decreases ostensibly. It should be considered that not enough time has elapsed since their publication for their influence to be effective in subsequent research.

#### 3.1.2. Distribution of Scientific Production

**Figure 3:**
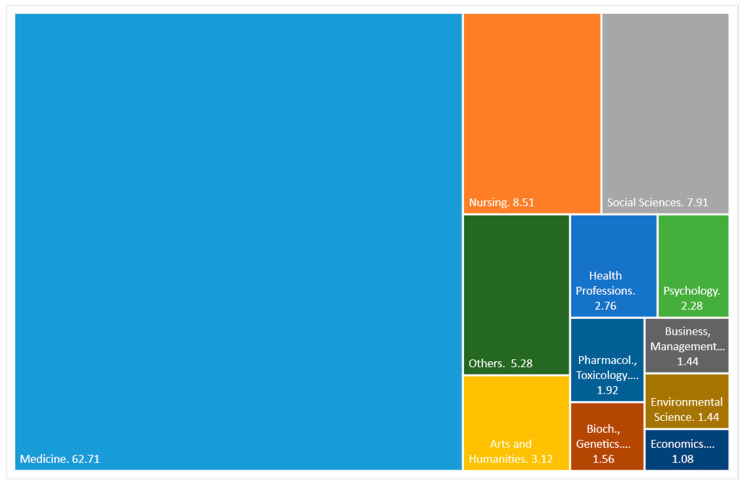
Documents by subject area (percentage).

Figure 3 shows the main areas on which Scopus classifies scientific production on PS and HP. The medicine theme prevails widely over the rest with 62.71%. Far behind, with 8.51% and 7.91%, are nursing and social sciences, respectively. The rest of the defined themes have less than 4% each. As we can see, the subject is mainly approached with merely medical criteria and not from an economic or investment management point of view, areas that, as a whole, do not reach 3%.

Table 2 displays the 11 most productive PS and HP journals. These journals published 20.13% (125 of 621) of the total number of articles included in this study, which shows that scientific activity in PS and HP is distributed in a large number of journals. The first four journals produced 55.2% of the top 11, and these journals were Social Science and Medicine, BMC Health Services Research, Health Policy, and BMJ Open, in order of productivity.

Table 2 exhibits other bibliometric indicators such as the average number of citations per year since the 1st published article, the average citation by paper, the year corresponding to the first published article, the year corresponding to the last published article, the Scimago Journal Rank (Quartile in 2019), and the h-index.

It is worth noting that the first journal in the ranking, with 19 articles, generated 644 citations, while the second in the ranking with only one fewer article generated 228 fewer citations than the first. The journal with the most citations is Social Science and Medicine. However, concerning the average number of citations per article, Health Affairs leads with 53 citations per article. Far behind is the leader in scientific productivity, with 33.89 citations per article.

An important point to highlight is that none of the journals included in the top 11 has scientific activity in this area in all the years analysed, the average being 15.54 years. To reduce the effect of the number of years of publication, the average citation per year was calculated from the first year of publication, where Social Science and Medicine maintain the lead with 40.25 citations per year, followed by BMC Health Services Research with 29.71, and in third place is Health Affairs with 21.20.

Regarding the h-index, Social Science and Medicine stands out widely from the rest with an h-index of 15. BMC Health Services Research follows it with 11 and Health Policy with 9. The fourth place is shared by Health Affairs and the International Journal of Healthcare Quality Assurance with an h-index of 8. Regarding the quartile of the SJR indicator, 8 of the 11 journals are in quartile 1, which means that patient satisfaction as the basis for decision-making is an appealing topic for high-impact journals.

A noteworthy fact is that 8 of the 11 journals included in the top 11 are from the United Kingdom, which means that this country is interested in researching patient satisfaction to formulate health policies. The rests are two European (one of Irish origin and one of Swiss origin) and one American.

#### 3.1.3. Countries, Institutions, Authors, and Papers

**Figure 4:**
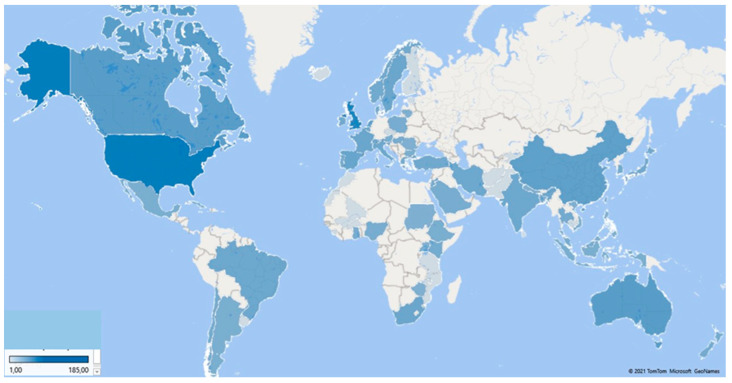
Worldwide publications on PS and HP.

Figure 4 illustrates a map of the countries that produce PS and HP articles, and Table 3 shows the data of the 11 most productive countries. It can be seen that most of the published articles are concentrated in the United States and the United Kingdom, with 185 and 144 published articles, respectively, which implies that authors from these two countries published 52.97% of PS and HP articles. It should be remembered that, in this analysis, a publication may represent more than one country because the authors’ affiliation institutions represent the publishing countries.

The next most productive are English-speaking countries or countries with a very high English proficiency according to the EF English Proficiency Index 2020 [79]: Canada with 43 articles, Australia with 42, and Germany with 40. This fact is not surprising because the report above indicates that English proficiency is related to the Global Talent Competitiveness Index, which measures a country’s ability to attract, develop, and retain talented people and invest in research and development. The importance of the English language in the scientific field dates back to the Industrial Revolution because those who promoted this movement used this language as it was their mother tongue (British and American). Consequently, those who wanted to learn about the advances had to learn the Anglo-Saxon language [80]. According to the United Nations report [81], the United States, the United Kingdom, and Germany are among the ten countries with the highest investment in research and development.

Analysing the total number of citations, the United States is ahead, followed by the United Kingdom and Canada. On the other hand, if the analysis is based on the number of citations per article, Canada is the first, followed by the Netherlands, the United Kingdom, and Germany. The number of citations evaluates the scientific quality for which these countries are considered most useful for science. A point to highlight is that countries such as Spain and China began scientific production on this subject 9 and 8 years, respectively, later than the countries that lead the ranking, so it is expected that the number of citations will grow in the future. By considering the h-index, the names of the leading countries are the same. In first place is the United Kingdom, second place the United States, third place the Netherlands, and Canada and Germany share fourth place. 

In the ranking of the 11 most productive countries, only five were productive during the 20 years studied. A relevant piece of information that can signify the diversity and growth of research on PS and HP is that the countries are on all of the continents. The United States and Canada represent America; Europe is represented by the United Kingdom, Germany, the Netherlands, Spain, France, and Sweden; Oceania accounts for Australia; China for Asia; and South Africa for Africa.

**Figure 5:**
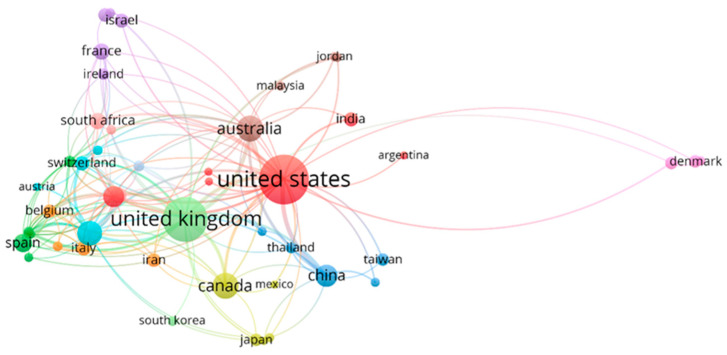
Network of cooperation based co-authorship between countries.

On the other hand, Figure 5 represents a co-authorship network based on international collaboration between countries with at least three articles published on PS and HP. The volume of the circles varies depending on the number of articles published. The colour corresponds to a cluster that encompasses each of the groups of countries. Twelve different groups can be observed.

The United States led the red cluster, representing a robust collaborative link with Argentina, Ethiopia, India, the Netherlands, and the Philippines. Spain heads the dark green group, and its major collaborating countries are all from the European continent (Bulgaria, Greece, Hungary, Norway, and Poland). China fronted the blue cluster, whose collaborators are from the same continent: Bangladesh, Hong Kong, Taiwan, and Thailand. For its part, the yellow cluster has representatives from the American continent (Canada and Mexico), Asia (Indonesia and Japan) and Africa (Nigeria). The dark purple group represents co-authorship from Brazil, France, Ireland, Israel, and Portugal.

On the other hand, the light blue comprises Austria, Chile, Germany, and Switzerland. Likewise, Belgium, Iran, Italy, and Slovenia are grouped in orange. In addition, the brown group is led by Australia, Joran, Malaysia, and Saudi Arabia. The light purple cluster, headed by Denmark, also includes Ghana and Turkey. The countries associated with the salmon colour are Kenya, South Africa, and Sweden. The light blue, orange, and brown groups are composed of co-authors representing four countries each; the light purple and salmon cluster by three countries, the light green by two countries, and the last set only by New Zealand.

Table 4 shows the ten countries that have contributed the most to scientific production through collaboration with other countries. The United States leads the ranking with 89 collaborations. China, the United Kingdom, Australia, and Canada are the countries that have collaborated the most with the United States in scientific production. Moreover, the country closest to the United States regarding the number of collaborators is the United Kingdom (85). The rest of the countries have much smaller collaborations: Germany (58), Australia (47), the Netherlands (38), and China (34). The last four positions in the top 10 have between 23 and 21 collaborators each. The United Kingdom is listed as a contributor to all countries in the top 10, as is the United States, though there is no colaboration with Belgium. The latter has a considerable number of citations, 373 for only nine published papers.

#### 3.1.4. Productivity of the Most Prolific Authors

Table 5 shows the 17 most relevant authors in the scientific literature on PS and HP. These authors represent thirteen academic institutions. The main characteristics include the number of articles, total citations, total citations by article, the year corresponding to the first published article, the year corresponding to the last published article, the average number of citations per year since the first published article, and the h-index, all of which are displayed in the table.

Considering the number of articles published and the h-index, we can divide the authors into two groups. The first comprising the two authors with six published articles and an h-index of 6. The second group, consisting of the remaining 15 authors, with three published articles each and an h-index of 3. Of the 17 authors, ten are of European origin, and six are from the United Kingdom. There are five authors of Israeli origin who participate in this ranking. These authors have three articles published in the period analysed, with 28 citations by authorship, which is possible because they are co-authors in the three scientific productions. Only two authors represent the American continent.

None of the authors of this ranking published during 2020, while 6 of the 17 published the last paper in 2019. In 2016, six of the 17 authors listed in Table 5 published their first article on PS and HP, and 15 of these authors published in the second part of the period analysed (2010–2020), which indicates that this line of research is booming.

The two most prolific authors are Bower, P. and Roland, M., affiliated with the Universities of Manchester and Cambridge, respectively. Both authors are from the United Kingdom. They have six published articles, an h-index of 6, their first publication in 2006 and their last in 2014. Bower, P. surpasses Roland, M. in the number of citations. The most popular work of both was “The GP patient survey for use in primary care in the national health service in the UK- development and psychometric characteristics”, published in 2009 and cited 94 times. This paper is one of the five articles that share authorship. These authors are also ranked first and second if we analyse the average number of citations per year since the first publication and the total number of citations.

The ranking is primarily led by Blendon, R.J. (86) regarding the number of citations per article with only three published papers. Far behind are Mead, N. (59.67), Cheraghi-Sohi, S. (53.33), and Kringos, D.S. (52.33), the latter also with only three articles published.

Authors with more than 100 citations began publishing on PS and HP in the first half of the period under review, except for Kringos D.S., who began publishing in 2011 and has 157 citations. 

**Figure 6:**
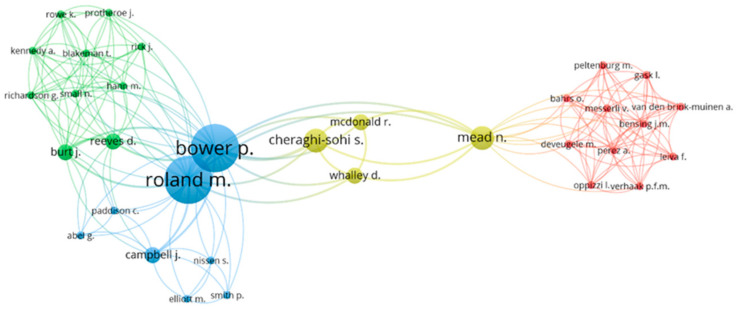
Network of cooperation based on co-authorship of the prominent authors.

Figure 6, made with the VOSviewer tool, represents the collaboration network among the principal authors. Close authors within the diagram are particularly collaborative, and the bubble size indicates the author’s relevance within the collaboration network. Only authors with works cited at least ten times have been taken into account. Four main collaborative groups have been found. The blue group is the only one with authors considered the most productive in this study (Bower, P. and Roland, M.). The country of the affiliate institution seems to determine the collaboration. The red cluster is made up of 11 authors belonging to institutions in the Netherlands. In the other three groups, all authors were affiliated with institutions of British origin.

The red circle comprises Bahrs, O., Bensing, J.M., Deveugele, M., Gask, L., Leiva, F., Messerli, V., Oppizzi, I., Peltenburg, M., Perez A., Van den Brink-Muinen, A., and Verhaak, P.F.M. The green circle includes ten authors: Burt, J., Blakeman, T., Hann, M. Kennedy, A., Protheroe, J., Reeves, D., Richardson, G., Rick, J., Rowe, K., and Small, N. On the other hand, the blue cluster comprises Abel, G. together with Bower, P., Campbell, J., Elliott, M., Nissen, S., Paddison, C., Roland, M., and Smith, P. Finally, the fourth cluster, in yellow, is formed by Mcdonals, R., Mead, N., and Whalley, D.

#### 3.1.5. Identification of the Main Research Institutions

Table 6 displays the 13 most productive PS and HP research institutions from 2000 to 2020, concentrated in 4 countries. It is worth noting that 61.54% are British. The United States and Canada have 15.385% each, and Israel has 7.69%. The table shows the data related to the citations and the first and last years of publication for each institution.

The University of Manchester is the institution that leads the ranking, with 14 articles and an h-index of 11. Three of the authors we previously named the most prolific belong to this University, accounting for 6 of the 14 articles published by this institution. Although it has a better h-index, it shares the number of articles with the London School of Hygiene and Tropical Medicine. It does not have better values than the other institutions concerning the rest of the parameters. The number of citations per article is 38.71, and the number of citations from the first year of publication is 27.10.

By considering the number of citations or the number of citations since the first year of publication, the leader in the ranking is the London School of Hygiene and Tropical Medicine. On the other hand, if the analysis is carried out from the number of citations per article, the leader is Imperial College London, with 62.58; this is also the institution to which the eighth-most prolific author belongs (see Table 5).

Except for Harvard Medical School, which first published in 2012, the institutions included in the ranking published in both analysis periods. Moreover, it is accurate to announce that 7 of the 13 published their last article on PS and HP in 2019. The University of Oxford and Harvard T.H. Chan School of Public Health are the institutions with the most extended history of research on public politics based on patient satisfaction.

London School of Hygiene and Tropical Medicine and Harvard T.H. Chan School of Public Health share second place in the h-index with a value of 9.

#### 3.1.6. Identification of the Most Cited Articles

Table 7 shows the 11 most cited titles during the analysed period, which is a relevant analysis as it reflects the most influential and popular titles in the scientific community. The year of publication, its authors, the total number of citations, and the average number of citations per year since its publication are indicated for each of them.

The success of the article “Systematic review of involving patients in the planning and development of healthcare” [23] is resounding, whether we analyse it from the point of view of the total number of citations (600) or if we analyse it as citations per year (31.58). It is followed in the ranking by “European patients” [82] views on the responsiveness of health systems and healthcare providers”, although with a 65% lower number approximately (214 citations).

It should be noted that Blendon, R.J, identified in Table 5 as one of the most prolific authors, is the author of one of the articles included in Table 7 within the ranking of the most cited. Its title is “Public trust in physicians—US medicine in international perspective” [83], and it corresponds to a publication from 2014 that has the highest number of citations per year (20.43) after the leading article. The rest of the articles in this Top 11 are not by authors considered more prolific.

An important fact that reveals the quality of the articles written by the author Coulter, A. is that his only two articles published on PS and HP during the study period are among the most cited. One of these was the one previously identified as the second most cited. The other is in tenth place with 122 citations [84].

### 3.2. Content Analysis

As indicated above, we divided the analysis period into two subgroups of 10 years each. The objective is to carry out a better analysis of the research evolution [65,85]. Considering it logical that research can change its study objectives over a period of 20 years and show an evolution, dividing the time horizon considered can give us a perspective of the researchers’ interest. The first period includes a total of 232 articles, while the second comprises 389.

**Figure 7:**
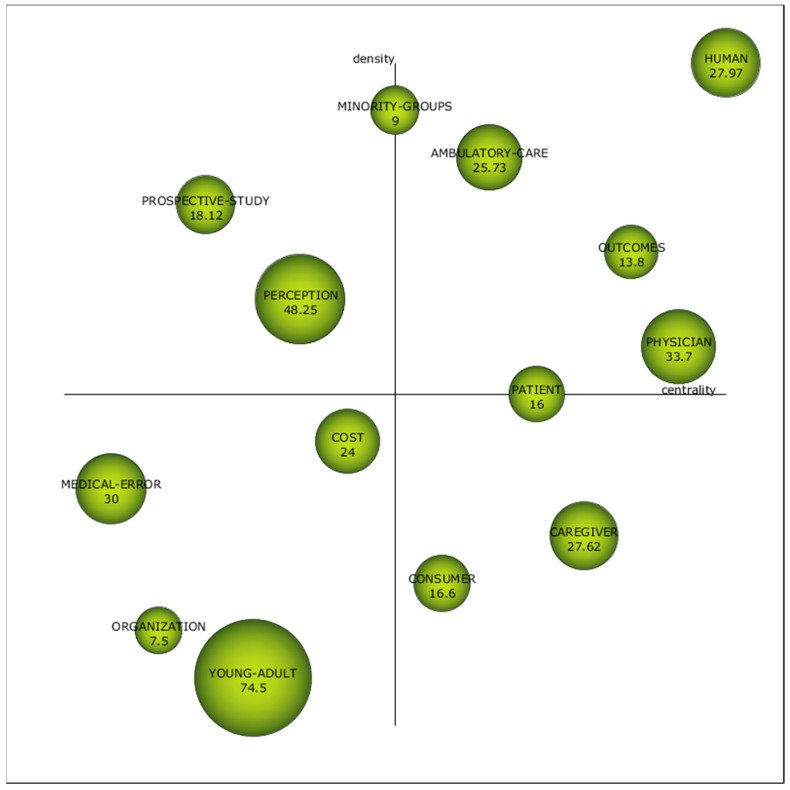
Strategic diagram of keywords based on documents-average citation from 2000–2010. Source: own elaboration.

Figure 7 exhibits the strategic diagram of the first sub-period (2000–2010). It illustrates four clearly defined motor keywords: “Human”, “Outcomes”, “Physician”, and “Ambulatory Care”, which, during this first period, were well developed and, therefore, were relevant in the research on PS and HP. Besides, two keywords are at the limit of being considered a motor keyword: “Patients”, whose density is not enough, and “Minority Groups” have a lower centrality than necessary to be regarded as proper motor keywords. These, also called driving themes, are in the upper-right quadrant, and they represent themes extensively developed and essential to shaping the scientific field [70].

The position of “Physician” is not surprising because most studies analyse patient satisfaction from a medical or nursing perspective, such as clinical preventive services [86], the communication skills of doctors [87], and technical quality [88,89], among others. In turn, all these aspects significantly influence the “Outcomes” obtained [90].

For its part, “Human” has total density and centrality, so we could affirm that it is a mature topic broadly connected with the rest of the keywords [91]. Another topic considered motor is “Ambulatory Care”, which has received particular attention from various authors [89,92,93,94].

In the reverse sector of the diagram, we find emerging or decaying keywords: “Medical Error”, “Organization”, ”Young-Adult”, and “Cost”, which are not the focus of current research, nor are they mature [95]. The subdivision of the period will allow us to observe if these issues acquire a better position or, on the contrary, end up disappearing.

Moreover, two basic themes, “Consumer” and “Caregiver”, and two peripheral themes, “Prospective Study” and “Perception”, were also identified. Words located in the upper left quadrant are not currently receiving attention but are potential research areas [96].

We can highlight the role of “Perception”, which is not yet sufficiently linked to the other research topics on PS and HP, although it is a highly developed topic.

The sphere’s size represents the number of citations per article, which is also indicated on each label. Table 8 complements Figure 7, showing the h-index, density, and centrality of the keywords.

The h-index of these keywords is led by two motor themes: “Human”, with 39, and “Physician”, with 17. The third place is shared, with an h-index of 7, a motor keyword (“Ambulatory-care”), and a basic one (“Caregiver”), which is logical because it deals with general or cross-cutting issues in the scientific field.

**Figure 8:**
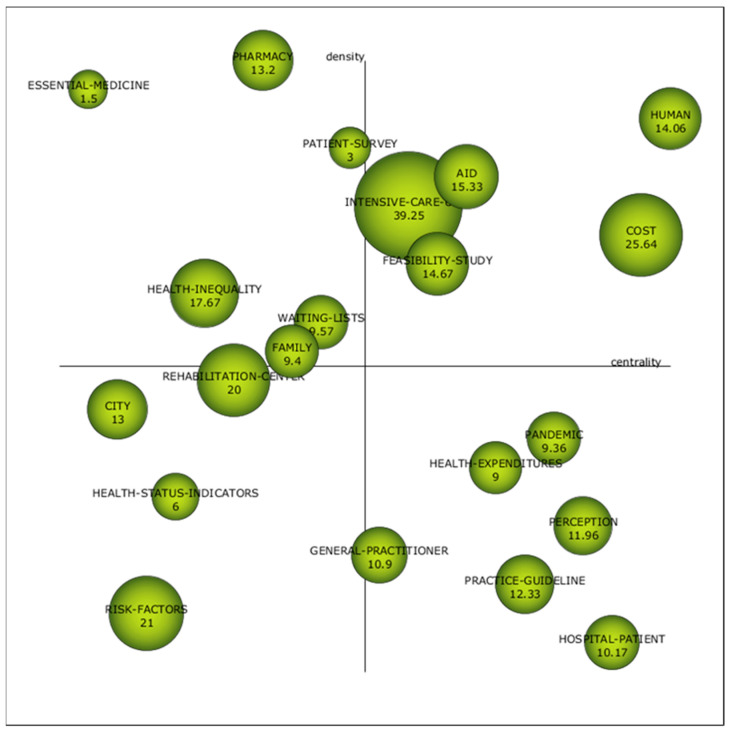
Strategic diagram of keywords based on documents-average citation from 2011–2020. Source: Own elaboration.

Figure 8 shows the strategic diagram of the second sub-period (2010–2020). It can be seen that the number of keywords has multiplied. Newly appeared are “Intensive Care”, “Feasibility Study”, and “Aid”. The authors identify points for improvement of public policies in different aspects of intensive care. On the one hand, Kasparian, N.A. seeks to develop better practices in paediatric intensive care [97]. On the other hand, Gunchan, P. studied the relationship between discharges against medical advice and the quality of public policies [98].

We can also find numerous feasibility studies within the bibliography that find relevant data for public policymakers [99,100,101,102].

“Human” continues to be a motor keyword with total centrality and 10% less density compared to the first period. “Cost” goes from being an emergent keyword to a motor keyword. 

For its part, “Perception” went from being a topic with high density and low centrality to becoming a basic topic, a keyword with high centrality and low density. Although clients’ perception has been studied since the 1950s [6,7], the economic crisis of 2008 [29,51,103,104] may have been a turning point in research on the perception of patients linked to the cost of healthcare.

For the other three topics in the emerging or declining quadrant in the first period, we can confirm they were declining keywords because they disappeared in the second period.

Table 9 complements Figure 8 by indicating the degree of density and centrality, h-index, and the number of documents and citations for each keyword. In this second period, the keyword with the best h-index continues to be “Human”, with 30. It is followed by the keyword “Perception”, which denotes the importance given to the evaluation of the patients’ opinion. Another keyword that emerges and reveals this to us is “Patient Survey” (keyword that goes hand in hand with “Perception” because it is a method to know it), which is in the peripheral quadrant at the limit with the quadrant of motor keywords. Table 9 shows relevant data for these terms; for example, “Perception” occurred in 25 papers and had 299 citations.

Besides “Perception”, we also observe the entry of the word “Pandemic” to the quadrant of basic keywords, a product of the health crisis caused by COVID-19. Some investigations are related to the advance in telemedicine that the COVID-19 forced [105,106].

The rest of the keywords in the lower right quadrant are compound words. “Health-Patient”, “Practice-Guideline”, and “Health-Expenditures” have a high centrality, which reveals the importance of these issues in the general development of public health policies based on patient satisfaction. Finally, “General-Practitioner” has a medium centrality, to the limit of becoming an emerging theme.

We found four emerging themes, that is, themes with low centrality and low density. They are “Risk-Factors”, “Health-Status-Indicators”, “City”, and “Rehabilitation-Centre”. None of them was found in the first period.

Finally, we highlight keywords with a high density that were highly developed independently in the scientific field analysed. They are ”Pharmacy”, “Essential-Medicine”, “Health Inequality”, “Waiting-List”, “Family”, and, the one already named, “Patient-Survey”.

**Figure 9:**
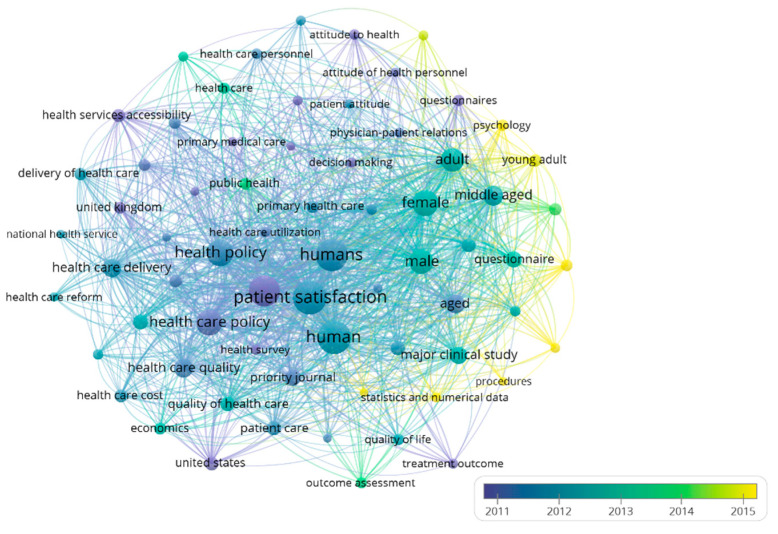
Evolution of leading keywords network based on co-occurrence (1954–2020). Own elaboration.

Figure 9 shows the evolution of keywords within the PS and HP research field, complementing the study of trends. For the analysis, only those words that appeared at least 40 times were taken. Blue indicates older terms used in the literature. Instead, the yellow colour represents terms that appeared more recently in the field of research under analysis.

During 2011, the articles were more related to patient satisfaction, healthcare quality, healthcare policy, and health services accessibility. During 2012, many keywords coincided with the previous year, such as the healthcare quality and public health policies. These words constitute a cluster that includes other keywords such as “National Health Service” or “Healthcare reform”.

In 2013, cross-sectional studies appeared as well as studies with gender distinction and outcomes assessment. In 2014, psychological aspects were incorporated into the evaluation of patient satisfaction to formulate public policies. In addition, the studies began to appear with statistical methodologies and numerical data.

## 4. Discussion

The objective of this study was to analyse research activity in the field of patient satisfaction as a basis for the formulation of public policies. We studied the temporal evolution of the theme from the point of view of keywords and the number of publications found. The most prolific authors and journals, collaborative relationships between countries, and scientific distribution were also analysed. Using the Scopus database, a sample of 621 articles published between 2000 and 2020 was obtained.

Although we could find that the first published articles were published in 1979 [107,108], scientific production on this subject began to flourish after 2000. Less than 11% of the total articles published correspond to the 1979–1999 period, which shows the interest generated after this time. This fact could be connected with the generation of the European Community Health Indicators (ECHI), which the European Union created to measure, among other things, the satisfaction of patients with the health system. The first part of the ECHI ended in 2001 [109]. The ECHI indicators arose to gather information that is not easy to obtain but useful for generating public policies [109,110].

Another turning point can be considered the financial crisis of 2008. After that year, 73.10% of the total scientific production was on PS and HP. The 2008 financial crisis caused a decrease in health budgets [51], so the study of satisfaction in these contexts became attractive. Analysis of patient satisfaction before and after the crisis is helpful to contribute to the formulation of public policies that improve the quality of the health system [111]. For example, considering that the length of stay is the primary determinant of the cost of hospitalisation, analyses will be carried out to reduce this stay without reducing the quality of care [112].

The main subject area is Medicine, followed by Nursing and Social Sciences, which is logical because health policy and patient satisfaction are framed within these large study groups. However, patient satisfaction from the point of view of health investment management and the application of resources is not widely covered. The following data can prove this: The business management area covers only 1.44% and the economic area 1.08%.

The two most productive authors are British and have six articles each. Bower P., belonging to the University of Manchester, is in first place, and Roland M, a member of the University of Cambridge, is second. These two authors are also leaders in the number of citations. However, the statehood belonging to the Harvard School of Public Health, Blendon R.J, is the author with the most extensive experience (13 years) and the highest value of citations per article (86). In this field of research, the most proliferating institution with the most significant impact is the University of Manchester, with 14 published articles and an h-index of 11. However, the institution with the highest number of citations is the London School of Hygiene and Tropical Medicine. The Imperial College London has the highest number of citations per article.

In order of importance, the five most productive countries are the United States, the United Kingdom, Canada, Australia, and Germany. All of them, except Canada, are also included in the list of the five most cooperative. The United States leads the position of both rankings with 185 articles published and 89 collaborations. On the contrary, the United Kingdom has the best quality measured according to the number of citations received, with an h-index of 33.

This study improves research on PS and HP because it allows us to visualise the state of scientific production and, above all, with the evolution of keywords, to identify possible future avenues of research. In this sense, we determined that the five most important topics studied in the current literature are: Intensive Care Units, Cost, Aid, Feasibility-study, and Human.

On the other hand, the strategic diagram allowed us to identify four emerging or decadent themes (Risk Factors, Rehabilitation Centre, City, and Health Status Indicators). For the study of the keywords, the period analysed was divided into two. However, in both sub-periods, the engine keyword with the highest h-index is the same: Human. Additionally, this keyword use leads in the number of documents, being 193 in the first ten years of study and 301 in the last ten years, which is logical because it is a global and generic issue. Perception is a theme that grew between the first period (4 documents) and the second period (25 papers). This contribution is relevant to the research because we can see that the patient's perception of perceived health services is increasingly considered when deciding what investments, expenses, and practices to carry out.

Of the five most productive journals, four are of British nationality. The first place is Social Science and Medicine, and the second is BMC Health Services Research, with 19 and 18 published articles, respectively. It was to be expected to find journals that deal with the subject of health in general terms. For this reason, other bibliometric studies on health services also find them among the most prolific [113]. In third place is an Irish journal that surpasses the previous ones in trajectory, being its first publication in 2001 and its last in 2019. Eight of the eleven most productive journals in 2019 belonged to quartile 1 in the Scimago Journal Rank (JCR).

Although no bibliometric studies were found on the formulation of public policies based on the patients’ satisfaction in the health system, an analysis of scientific production was found on closely related topics, such as the quality of the health system [54] and the application of marketing to public services [114].

This article aims to show which institutions, authors, and countries produce science in the field of public policy formulation subject to patient satisfaction. The use of marketing concepts, such as satisfaction, is reaching a certain maturity in the public sector, and more specifically, in health [114]. The ultimate goal is for policymakers to bring down the scientists’ concepts to reality to make better decisions that positively impact the population’s quality of life.

This research has some limitations. The Scopus database was used. Although most of the articles in the WoS database are in Scopus, it would be interesting to develop this analysis based on WoS to verify that the results obtained are similar. A bibliometric study on sustainability and public health that compares both databases found that Scopus, until 2013, was the leader in the volume of articles. However, from 2013 to 2017, the concentration was similar in both databases [115]. WoS performs a comprehensive content filter based on citation data, posting standards and expert judgments [68].

On the other hand, Google Scholar is advancing in quality, so conducting a study on this platform would also be interesting. Google Scholar is limited to publications in scientific journals and includes communications and presentations to congresses, theses, seminars, and other academic works that can profoundly contribute to the field studied [116]. Another future research could be to focus only on public policies solely focused on the financing of health services or their quality. In addition, a timed h-index study could be carried out to verify that the authors considered to be the most prolific continue to be so today or if their h-index, calculated traditionally, is high as a consequence of successful but old publications [117].

## 5. Conclusions

We carried out a bibliometric study based on 621 articles from the Scopus database on PS and HP published between 2000 and 2020. The study revealed that the scientific production on the subject was not significant in terms of quantity in previous years. However, from the year 2000, production began to accelerate. We estimate the latter to be due to the appearance of the ECHI indicators. Therefore, starting in 2010, a greater preoccupation can be observed in studying the perception of patients.

We believe that researchers must understand the political processes in health matters. At the same time, politicians have to communicate with researchers because it will be the only way scientific discoveries can be applied in real life and improve the population's health and quality of life.

Finally, we want to underline that two potent tools have been used: VOSviewer and SciMAT. In the bibliography, it can be observed that, in general, only a single bibliometric tool is used.

## Figures and Tables

**Table 1:** Summary of data.

Year	A	AU	AU/A	C	TC	TC/A	TC/AU
2000–2010	232	819	3.53	50	6455	27.82	7.88
2011–2020	389	1884	4.84	77	5267	13.54	2.80
Total period	621	2672	4.30	84	11722	18.88	4.39

A: number of articles; AU: number of authors; AU/A: number of authors by article; C: number of countries; TC: total citations in the articles; TC/A: total citations per article; TC/AU: total citations per author. Source: own elaboration.

**Table 2:** The top 11 most productive journals on PS and HP from 2000–2020.

Journal	A	C	TC	TC/A	1st A	Last A	TC/Y	SJR(Q)	H-Index
Social Science and Medicine	19	U.K.	644	33.89	2005	2020	40.25	1.944(Q1)	15
BMC Health Services Research	18	U.K.	416	23.11	2006	2020	29.71	0.995(Q1)	11
Health Policy	18	Ireland	294	16.33	2001	2019	14.70	1.097(Q1)	9
BMJ Open	14	U.K.	76	5.43	2015	2020	12.67	1.247(Q1)	5
Health Policy and Planning	9	U.K.	161	17.89	2005	2019	10.06	1.620(Q1)	6
Int. Journal of Environmental Research and Public Health	9	Switzerland	42	4.67	2015	2020	7.00	0.739(Q2)	5
Int. Journal of Health Planning and Management	9	U.K.	37	4.11	2006	2020	2.47	0.537(Q2)	4
Health Affairs	8	U.S.	424	53.00	2001	2016	21.20	3.766(Q1)	8
British Journal of General Practice	7	U.K.	63	9.00	2004	2019	3.71	0.938(Q1)	6
Health Services Research	7	U.K.	105	15.00	2001	2019	5.25	1.623(Q1)	5
Int. Journal of Healthcare Quality Assurance	7	U.K.	180	25.71	2000	2018	8.57	0.340(Q2)	8

A: number of articles; C: country; TC: total citations; TC/A: total citations by article; 1st A: year corresponding to first published article; Last A: year corresponding to last published article; TC/Y: average number of citations per year since the 1st published article; SJR(Q): Scimago Journal Rank; Q1 and Q2 are the first and second Quartile, respectively, in 2019; h-index: Hirsch in this topic. Source: own elaboration.

**Table 3:** The top 11 most productive countries on PS and HP research.

Country	A	TC	TC/A	1st A	Last A	TC/Y	H-Index
United States	185	3478	18.80	2000	2020	165.62	30
United Kingdom	144	3787	26.30	2000	2020	180.33	33
Canada	43	1386	32.23	2000	2020	66.00	18
Australia	42	762	18.14	2000	2020	36.29	16
Germany	40	884	22.10	2003	2020	49.11	18
China	30	454	15.13	2008	2020	34.92	11
Netherlands	27	837	31.00	2000	2020	39.86	19
Spain	18	190	10.56	2009	2020	15.83	9
France	15	233	15.53	2003	2017	12.94	8
South Africa	15	236	15.73	2005	2019	14.75	9
Sweden	15	245	16.33	2000	2018	11.67	10

A: number of articles; TC: total citations; TC/A: total citations by article; 1st A: year corresponding to first published article; Last A: year corresponding to last published article; TC/Y: average number of citations per year since the 1st published article; h-index: Hirsch in this topic. Source: Own elaboration.

**Table 4:** Top 10 most cooperative countries and main collaborators.

Country	A	C	NC	Main Collaborators
U.S.	183	3296	89	China, U.K., Australia, Canada
U.K.	144	3865	85	Australia, U.S., Canada, China
Germany	39	879	58	Netherlands, U.K., Switzerland, U.S.
Australia	42	762	47	UK, US, China
Netherlands	27	826	38	Germany, U.S., Belgium, UK.
China	30	454	34	U.S., Australia, U.K., Germany
Canada	42	1212	23	U.S., U.K., Germany, Australia
Belgium	9	373	22	Netherlands, U.K., South Africa
Norway	7	127	22	U.K., Germany, Australia, U.S.
Switzerland	11	112	21	Germany, U.K., Australia, US.

A: number of articles, C: number of citations, NC: number of collaborations. Source: Own elaboration.

**Table 5:** The top 17 most productive authors on PS and HP research.

Author	A	TC	TC/A	1st A	Last A	TC/Y	H-Index	Country	Affiliation
Bower, P.	6	296	49.33	2006	2014	19.73	6	U.K.	University of Manchester
Roland, M	6	270	45.00	2006	2014	18.00	6	U.K.	University of Cambridge
Balicer, R.D.	3	28	9.33	2016	2019	5.60	3	Israel	Clalit Research Institute
Blendon, R.J.	3	258	86.00	2001	2014	12.90	3	U.S.	Harvard School of Public Health
Cheraghi-Sohi, S.	3	160	53.33	2006	2008	10.67	3	U.K.	University of Manchester
Davidovitch, N.	3	28	9.33	2016	2019	5.60	3	Israel	Ben-Gurion University of the Negev
Ernstmann, N.	3	44	14.67	2011	2014	4.40	3	Germany	Institute for Medical Sociology
Greenfield, G.	3	28	9.33	2016	2019	5.60	3	U.K.	Imperial College London
Hekselman, I.	3	28	9.33	2016	2019	5.60	3	Israel	Clalit Mushlam Health Insurance
Kringos, D.S.	3	157	52.33	2011	2016	15.70	3	Netherlands	University of Amsterdam
Mead, N.	3	179	59.67	2000	2007	8.52	3	U.K.	University of Manchester
Pfaff, H.	3	44	14.67	2011	2014	4.40	3	Germany	University of Cologne
Pliskin, J.S.	3	28	9.33	2016	2019	5.60	3	Israel	Ben-Gurion University of the Negev
Ryan, M.	3	61	20.33	2006	2014	4.07	3	U.K.	University of Aberdeen
Shi, L.	3	60	20.00	2008	2015	4.62	3	U.S.	Johns Hopkins University
Shmueli, L.	3	28	9.33	2016	2019	5.60	3	Israel	Ben-Gurion University of the Negev
Strech, D.	3	102	34.00	2010	2018	9.27	3	Germany	Berlin Institute of Health

A: number of articles; TC: total citations; TC/A: total citations by article; 1st A: year corresponding to first published article; Last A: year corresponding to last published article; TC/Y: average number of citations per year since the 1st published article; h-index: Hirsch in this topic. Source: Own elaboration.

**Table 6:** The top 13 most productive institutions on PS and HP research.

Institution	Country	A	TC	TC/A	1st A	Last A	TC/Y	H-Index
The University of Manchester	U.K.	14	542	38.71	2001	2016	27.10	11
London School of Hygiene and Tropical Medicine	U.K.	14	770	55.00	2002	2018	40.53	9
University of Toronto	Canada	13	483	37.15	2003	2019	26.83	8
Imperial College London	U.K.	12	751	62.58	2002	2019	39.53	8
University of Oxford	U.K.	10	89	8.90	2001	2019	4.45	6
Harvard T.H. Chan School of Public Health	U.S.	10	387	38.70	2001	2019	19.35	9
Harvard Medical School	U.S.	8	194	24.25	2012	2019	21.56	6
King’s College London	U.K.	8	100	12.50	2001	2018	5.00	7
University College London	U.K.	8	150	18.75	2008	2019	11.54	5
University of Calgary	Canada	7	79	11.29	2002	2018	4.16	4
London School of Economics and Political Science	U.K.	7	256	36.57	2008	2015	19.69	6
University of Aberdeen	U.K.	7	179	25.57	2006	2016	11.93	6
Ben-Gurion University of the Negev	Israel	7	57	8.14	2005	2019	3.56	5

A: number of articles; TC: total citations; TC/A: total citations by article; 1st A: year corresponding to first published article; Last A: year corresponding to last published article; TC/Y: average number of citations per year since the 1st published article; h-index: Hirsch in this topic. Source: Own elaboration.

**Table 7:** The top 11 most cited articles on PS and HP research.

Title	Author/s	Journal	TC	Year	TC/Year
Systematic review of involving patients in the planning and development of healthcare	Crawford M.J., Rutter D., Manley C., Weaver T., Bhui K., Fulop N., Tyrer P.	British Medical Journal	600	2002	31.58
European patients’ views on the responsiveness of health systems and healthcare providers	Coulter A., Jenkinson C.	European Journal of Public Health	214	2005	13.38
The use of patient-reported outcomes (PRO) within comparative effectiveness research: Implications for clinical practice and healthcare policy	Ahmed S., Berzon R.A., Revicki D.A., Lenderking W.R., Moinpour C.M., Basch E., Reeve B.B., Wu A.W.	Medical Care	172	2012	19.11
Client satisfaction and quality of healthcare in rural Bangladesh	Aldana J.M., Piechulek H., Al-Sabir A.	Bulletin of the World Health Organization	153	2001	7.65
Public trust in physicians–US medicine in international perspective	Blendon R.J., Benson J.M., Hero JO.	New England Journal of Medicine	143	2014	20.43
Cannabis for therapeutic purposes: Patient characteristics, access, and reasons for use	Walsh Z., Callaway R., Belle-Isle L., Capler R., Kay R., Lucas P., Holtzman S.	International Journal of Drug Policy	143	2013	17.88
New federal policy initiatives to boost health literacy can help the nation move beyond the cycle of costly 'crisis care	Koh H.K., Berwick D.M., Clancy C.M., Baur C., Brach C., Harris L.M., Zerhusen E.G.	Health Affairs	143	2012	15.89
Subjective unmet need and utilisation of healthcare services in Canada: What are the equity implications?	Allin S., Grignon M., Le Grand J.	Social Science and Medicine	136	2010	12.36
Private healthcare quality: Applying a SERVQUAL model	Butt MM, de Run EC.	International Journal of Healthcare Quality Assurance	124	2010	11.27
What do patients and the public want from primary care?	Coulter A.	BMJ	122	2005	7.63
Provider continuity in family medicine: does it make a difference for total healthcare costs?	De Maeseneer J.M., De Prins L., Gosset C., Heyerick J.	Annals of family medicine	122	2003	6.78

TC: total citations; TC/Year: average number of citations per year since the article was published. Source: Own elaboration.

**Table 8:** The characteristic of the strategic diagram topics from 2000 to 2010.

Topics	Documents	H-Index	Citations	Centrality	Density
Human	193	39	5399	1.00	1.00
Minority-groups	2	2	18	0.5	0.93
Outcomes	5	4	69	0.86	0.71
Ambulatory-care	11	7	283	0.64	0.86
Prospective-study	8	6	145	0.21	0.79
Perception	4	4	193	0.36	0.64
Cost	5	5	120	0.43	0.43
Caregiver	8	7	221	0.79	0.29
Physician	27	17	910	0.93	0.57
Patient	9	5	144	0.71	0.50
Consumer	5	4	83	0.57	0.21
Organization	2	1	15	0.14	0.14
Young-adult	2	2	149	0.29	0.07
Medical-error	1	1	30	0.07	0.36

H-index: Hirsch in this topic. Source: own elaboration.

**Table 9:** The characteristic of the strategic diagram topics from 2011 to 2020.

Topics	Documents	H-Index	Citations	Centrality	Density
Human	301	30	4232	1.00	0.90
Pharmacy	5	3	66	0.33	1.00
Aid	3	3	46	0.67	0.81
Patient-survey	4	3	12	0.48	0.86
Intensive-care-unit	4	3	157	0.57	0.76
Feasibility-study	9	5	132	0.62	0.67
Family	5	4	47	0.38	0.52
Cost	11	7	282	0.95	0.71
Waiting-lists	7	4	67	0.43	0.57
Pandemic	11	6	103	0.81	0.38
Perception	25	10	299	0.86	0.24
General-practitioner	10	6	109	0.52	0.19
Practice-guideline	12	7	148	0.76	0.14
Health-expenditures	4	4	36	0.71	0.33
Hospital-patient	6	4	61	0.90	0.05
Health-inequality	3	3	53	0.24	0.62
Rehabilitation-centre	2	2	40	0.29	0.48
City	2	2	26	0.10	0.43
Health-status-indicators	2	2	12	0.19	0.29
Essential-medicine	2	1	3	0.05	0.95
Risk-factors	1	1	21	0.14	0.10

H-index: Hirsch in this topic. Source: own elaboration.

## Data Availability

The data presented in this study are available on request from the corresponding author.
